# Compound Heterozygosity for Novel Truncating Variants in the *LMOD3* Gene as the Cause of Polyhydramnios in Two Successive Fetuses

**DOI:** 10.3389/fgene.2019.00835

**Published:** 2019-09-13

**Authors:** Ye Wang, Caixia Zhu, Liu Du, Qiaoer Li, Mei-Fang Lin, Claude Férec, David N. Cooper, Jian-Min Chen, Yi Zhou

**Affiliations:** ^1^Fetal Medicine Center, Department of Obstetrics and Gynecology, The First Affiliated Hospital of Sun Yat-Sen University, Guangzhou, China; ^2^Department of Ultrasonic Medicine, The First Affiliated Hospital of Sun Yat-Sen University, Guangzhou, China; ^3^Jiangmen Central Hospital, Affiliated Jiangmen Hospital of Sun Yat-Sen University, Jiangmen, China; ^4^EFS, Univ Brest, Inserm, UMR 1078, GGB, Brest, France; ^5^CHU Brest, Service de Génétique, Brest, France; ^6^Institute of Medical Genetics, School of Medicine, Cardiff University, Cardiff, United Kingdom

**Keywords:** *LMOD3*, nemaline myopathy, polyhydramnios, prenatal diagnosis, truncating variant, whole exome sequencing

## Abstract

Polyhydramnios is sometimes associated with genetic defects. However, establishing an accurate diagnosis and pinpointing the precise genetic cause of polyhydramnios in any given case represents a major challenge because it is known to occur in association with over 200 different conditions. Whole exome sequencing (WES) is now a routine part of the clinical workup, particularly with diseases characterized by atypical manifestations and significant genetic heterogeneity. Here we describe the identification, by means of WES, of novel compound heterozygous truncating variants in the *LMOD3* gene [i.e., c.1412delA (p.Lys471Serfs*18) and c.1283dupC (p.Gly429Trpfs*35)] in a Chinese family with two successive fetuses affected with polyhydramnios, thereby potentiating the prenatal diagnosis of nemaline myopathy (NM) in the proband. *LMOD3* encodes leiomodin-3, which is localized to the pointed ends of thin filaments and acts as a catalyst of actin nucleation in skeletal and cardiac muscle. This is the first study to describe the prenatal and postnatal manifestations of *LMOD3*-related NM in the Chinese population. Of all the currently reported NM-causing *LMOD3* nonsense and frameshifting variants, c.1412delA generates the shortest truncation at the C-terminal end of the protein, underscoring the critical role of the WH2 domain in LMOD3 structure and function. Survey of the prenatal phenotypes of all known *LMOD3*-related severe NM cases served to identify fetal edema as a novel presenting feature that may provide an early clue to facilitate prenatal diagnosis of the disease.

## Introduction

Polyhydramnios, an excess of amniotic fluid in the amniotic sac, is diagnosed if the single deepest pocket (SDP) is of ≥ 8 cm or the amniotic fluid index (AFI) is of ≥ 25 cm (Moore and Cayle, 1990; Magann et al., 2007). It is present in 1–3% of pregnancies (Maymon et al., 1998; Biggio et al., 1999; Magann et al., 2007) and may occur as a consequence of both environmental (e.g., perinatal exposure to TORCH infections, maternal gestational and pregestational diabetes) and genetic factors (Bartha et al., 2003; Keshavarz et al., 2005; Touboul et al., 2007; Kishore et al., 2011; Kollmann et al., 2014). Indeed, a search for polyhydramnios in the Human Phenotype Ontology database (https://hpo.jax.org/app/browse/term/HP:0001561) yielded 204 different conditions and 143 different genes. Nearly one half of all pregnancies with polyhydramnios are associated with varying degrees of fetal abnormality, including fetal death (Kollmann et al., 2014). Therefore, it is extremely important to identify the genetic causes of polyhydramnios in affected families with a view to providing genetic counselling and prenatal diagnosis for subsequent pregnancies.

With the decreasing cost of next generation sequencing, whole exome sequencing (WES) has been increasingly employed as an important diagnostic tool for clinical purposes (Boycott et al., 2013; Lee et al., 2014; Posey et al., 2017), especially in the case of diseases characterized by atypical manifestations and significant levels of genetic heterogeneity (Ku et al., 2012). Here we describe the use of WES to identify the genetic cause of polyhydramnios in two successive fetuses in a Chinese family.

## Case Presentation

**Figure 1:**
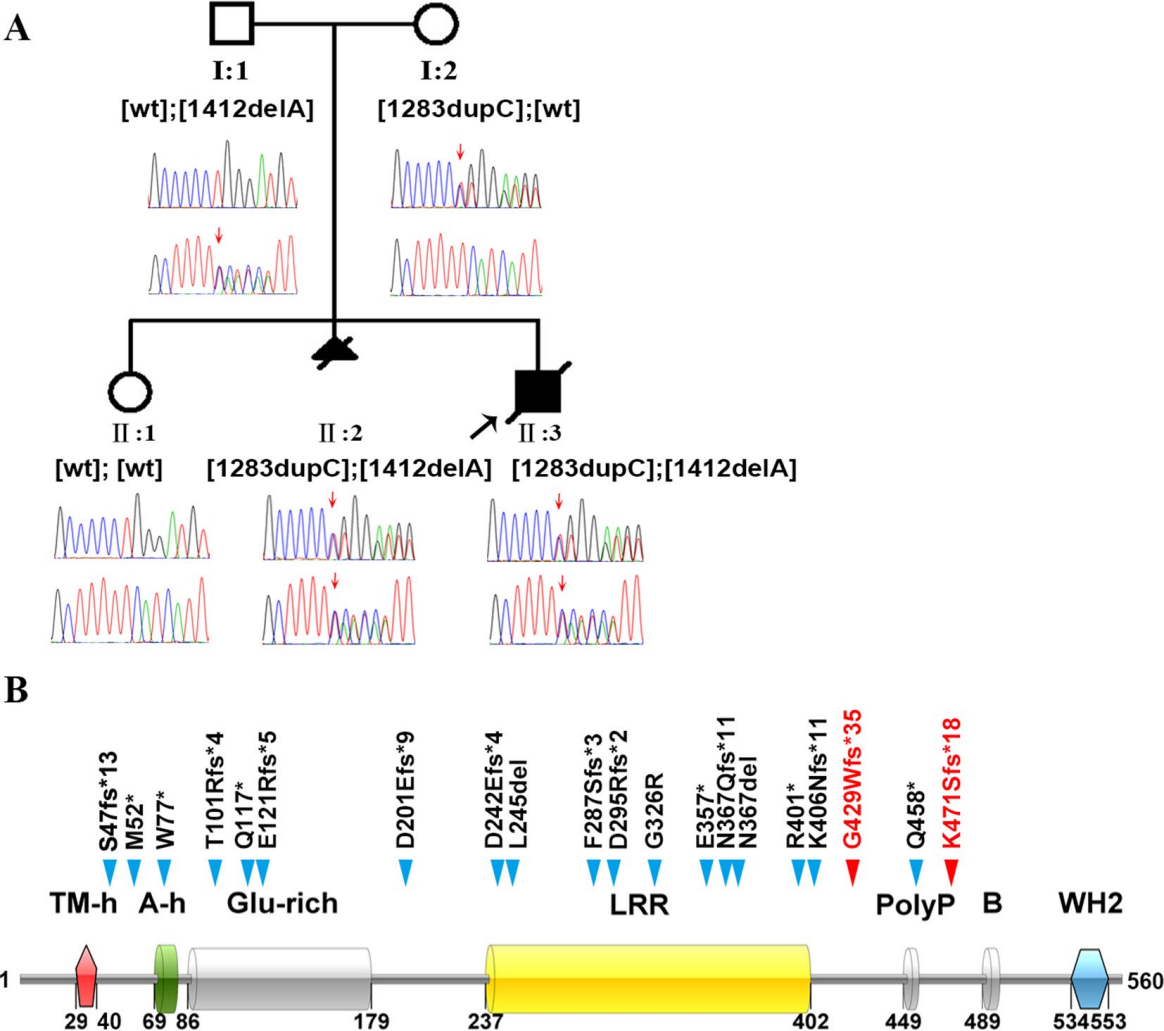
Identification of the genetic basis of polyhydramnios in a Chinese family. **(A)** Family pedigree and DNA sequencing results. Filled triangle with oblique line indicates the fetus with polyhydramnios terminated by therapeutic abortion. Filled square with oblique line indicates the affected boy who died after birth. Arrow indicates the proband. Open symbols indicate clinically unaffected family members. *LMOD3* genotypes are provided for all subjects. Red arrows indicate the mutation positions. wt, wild-type. **(B)** Illustration of the LMOD3 structure and all currently reported severe NM-causing *LMOD3* variants. The two novel variants identified in the current study are highlighted in red. Previous reported variants (in black) were from (Yuen et al., 2014; Berkenstadt et al., 2018; Michael et al., 2019). See **Supplementary Table S1** for variant descriptions at the nucleotide sequence level.

**Figure 2:**
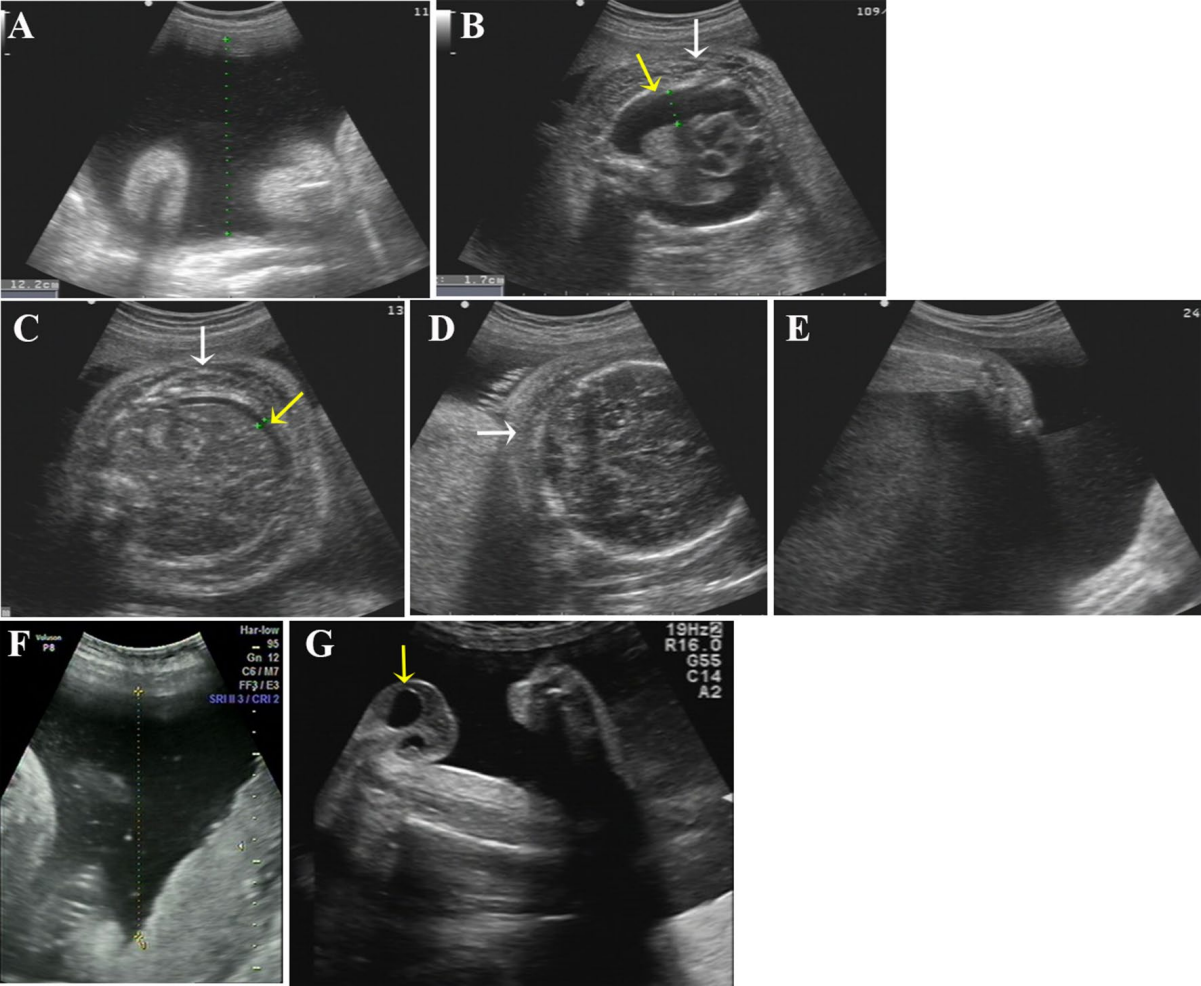
Ultrasound photographs of the 2 fetuses with polyhydramnios. **(A–E)** Ultrasound images of the first affected fetus (II-2) at 34 GW. Two-dimensional ultrasound showing polyhydramnios (amniotic fluid deep, AFD, 12.2 cm) **(A)** and fixed limbs **(E)**. Bilateral pleural effusion (**B**, yellow arrow), ascites (**C**, yellow arrow), skin edema of chest (**B**, white arrow), abdomen (**C**, white arrow), and scalp (**D**, white arrow) were detected by ultrasound. **(F, G)** Ultrasound pictures of the second affected nemaline myopathy fetus (II-3) at 33 GW. Two-dimensional ultrasound revealed polyhydramnios (amniotic fluid deep, AFD, 11.5cm) **(F)** and hydrocele of testis **(G)** (yellow arrow).

A 35-year-old woman was referred to our centre at the First Affiliated Hospital of Sun Yat-Sen University after her third fetus (II:3; **Figure 1A**) had been found to have polyhydramnios (SDP of 9.5 cm and an AFI of 30.7 cm) and hydrocele of testis at 29 gestational weeks (GW). Prior to this, her second fetus (II:2) had presented with polyhydramnios and generalized edema (pleural effusion, ascites, skin edema of the chest, abdomen, and scalp) at 34 GW (**Figures 2A–D**) and was then terminated at 36 GW. Her first child (II:1) is a healthy girl, now aged 6 years. Both parents were of south Chinese origin, healthy and non-consanguineous. Exposure to known mutagenic or teratogenic agents during pregnancy was not reported.

In the case of II:2, only standard G-banding karyotyping (using cord blood cells taken at 35 GW) followed by chromosomal microarray analysis were performed at the time, yielding negative findings. In the case of fetus II:3, molecular genetic analysis including WES was performed at 31 GW. The WES results were returned to us 6 weeks later, confirming a diagnosis of nemaline myopathy (NM) (see below). Genetic counselling was provided to the couple, who opted to continue the pregnancy. The boy was born by full-term vaginal delivery at 37 GW; his Apgar scores were 5 and 6 (normal, 10) at 1 and 5 min of life, respectively. He showed stiffness of limbs, little movement and scrotal swelling, and died of respiratory failure 2 days after birth.

Clinical findings in the two affected fetuses (II:2 and II:3) are illustrated in **Figure 2** and summarized in **Table 1**.

**Table 1:** Summary of prenatal feature in 24 *LMOD3* mutation-positive cases of nemaline myopathy.

Case	Reference	Gender	Ethnicity	Polyhydramnios	Decreased fetal movements	Arthrogryposis/contracture	Fractures	Fetal edema	Other anomalies	Preterm delivery	Age at death (or current age if alive)	*LMOD3* genotype^†^
1	Yuen et al., 2014	F	Algerian	+	–	+	+	–		+	neonatal	p.S47fs*13 homozygote
2		M	Belgian	–	+	+	–	–		–	10 months	p.S47fs*13 homozygote
3a		F	Portuguese	+	+	+	–	–		–	neonatal	p.M52* homozygote
3b		F	Portuguese	+	–	+	–	–		–	1 months, alive	
4		F	Japanese	+	+	–	–	–		–	2 months, alive	p.[T101Rfs*4]; [D201Efs*9]
5		F	Japanese	+	+	+	–	+	Subdural hematoma	+	10 months, alive	p.Q117* homozygote
6		F	Japanese	+	+	–	–	–	microcephaly	–	1 year, alive	p.[Q117*]; [K406Nfs*11]
7		F	Italian	+	+	+	–	–		+	4 months	p.F287Sfs*3 homozygote
8		M	Ecuadorianmn	+	+	+	+	–		+	6 weeks	p.[G326R]; [Q458*]
9		M	Swedish	+	–	+	–	–		–	5 months	p.E357* homozygote
10		M	Afghan	–	+	+	–	–		+	neonatal	p.N367Qfs*11 homozygote
11		M	Afghan	–	–	+	–	–		+	2 months	p.N367Qfs*11 homozygote
12		F	Pakistani	+	–	–	–	–		–	3 months	p.N367Qfs*11 homozygote
13		F	Pakistani	+	+	–	–	–		–	neonatal	p.N367Qfs*11 homozygote
14		F	Australian	+	+	–	–	–		–	10 years, alive	p.[N367del]; [R401*]
15		F	Australian	+	–	–	–	–		–	4 years, alive	p.[N367del]; [R401*]
16a	Abbott et al., 2017	F	Turkish	–	+	–	+	+		+	neonatal	p.S47fs*13 homozygote
16b		F	Turkish	–	+	–	+	–		+	2 months	
16c		F	Turkish	+	–	–	+	+		+	neonatal	
17	Berkenstadt et al., 2018	F	Moroccan	+	+	+	–	–		NE	induced abortion	p.[E121Rfs*5]; [L245del]
18	Michael et al., 2019	F	Turkish	+	+	+	–	+	atrial septum defect	–	8 years, alive	p.D295Rfs*2 homozygote
19		M	NE	+	+	+	–	–	atrial septum defect	–	5 months	p.E357* homozygote
20a	This study	Unknown	Chinese	+	+	–	–	+		NE	induced abortion	p.[G429Wfs*35]; [K471Sfs*18]
20b		M	Chinese	+	+	–	–	+		–	neonatal	
**Total**				**19/24** **(79.2%)**	**17/24** **(70.8%)**	**13/24** **(54.1%)**	**5/24** **(20.8%)**	**6/24** **(25.0%)**		**9/22** **(40.9%)**		

+, present; –, absent; F, female; M, male; NE, not evaluated.

^†^See **Supplementary Table S1** for variant descriptions at the nucleotide sequence level.

## Materials and Methods

### Subjects

This study was carried out in accordance with the recommendations of “ethical regulations of biomedical research involving humans, Ethics Committee of the First Hospital affiliated to Sun Yat-sen University” with written informed consent from all subjects. All subjects gave written informed consent in accordance with the Declaration of Helsinki. The protocol was approved by the “Ethics Committee of the First Hospital affiliated to Sun Yat-sen University”. Prenatal diagnosis was undertaken through ultrasound-guided umbilical cord blood puncture in accordance with standard practice.

### Karyotype and Chromosomal Microarray Analysis

Standard G-banding karyotyping was performed according to standard laboratory procedures. The chromosomal microarray experiments were conducted using the high-resolution Affymetrix CytoScan HD arrays (Affymetrix Inc., Santa Clara, CA) according to the manufacturer’s protocols. Detected copy number variants were evaluated for clinical significance by comparing them with data in the scientific literature and publicly available databases: UCSC (Ku et al., 2012), DECIPHER database (https://decipher.sanger.ac.uk/), Database of Genomic Variants (DGV, http://dgv.tcag.ca/dgv/app/home), the International Standards for Cytogenomic Arrays (ISCA, https://www.iscaconsortium.org/), and Online Mendelian Inheritance in Man (OMIM, https://www.omim.org/). The results were then analyzed by the Chromosome Analysis Suite software version 3.3.0; the reporting threshold of copy number variants was set at 10 kb, with marker count at over 50, as previously reported (Wang et al., 2015).

### Whole Exome Sequencing (WES)

WES was performed in fetus II:3 and its parents. Genomic DNA was randomly fragmented and then purified by means of the magnetic particle method. Sequences were captured by Agilent SureSelect version 4 (Agilent Technologies, Santa Clara, CA) according to the manufacturer’s protocols. After enrichment and purification, the DNA libraries were sequenced on a NextSeq500 sequencer following the manufacturer’s instructions (Illumina, San Diego). The reads were aligned to hg19/GRCh37.p10 using the Burrows-Wheeler Aligner (version 0.59) (Li and Durbin, 2009). Base quality recalibration and local realignment of the Burrows-Wheeler aligned reads were then processed by means of GATK IndelRealigner (https://software.broadinstitute.org/gatk/documentation/tooldocs/current/org_broadinstitute_gatk_tools_walkers_indels_IndelRealigner.php) and GATK BaseRecalibrator (https://software.broadinstitute.org/gatk/documentation/tooldocs/current/org_broadinstitute_gatk_tools_walkers_bqsr_BaseRecalibrator.php), respectively. Single nucleotide variations and small indels were revealed by the GATK UnifiedGenotyper (https://software.broadinstitute.org/gatk/documentation/tooldocs/current/org_broadinstitute_gatk_tools_walkers_genotyper_UnifiedGenotyper.php). Variants were finally annotated employing the Consensus Coding Sequences Database at the National Centre for Biotechnology Information (https://www.ncbi.nlm.nih.gov/CCDS/).

Putative causal variants were sought in an unbiased and hypothesis-free manner. Trio sample strategy was applied to remove the bias that may arise by proband-only sequencing. Literature and population databases were used for variant annotation, including 1000 Genomes, dbSNP, GenomAD, Clinvar, HGMD, and OMIM. The synonymous and common SNPs (MAF > 5%) were filtered out, and rare variants with high confidence were considered as disease-causing candidates for further genetic evaluation. Multiple computational algorithms were applied to assist the genetic evaluation of pathogenicity, including SIFT, Polyphen-2, and MutationTaster. Variants occurring in known phenotype-causing or -associated genes as well as in candidate genes selected on the basis of known biological, physiological or functional relevance to phenotype were considered with priority. The interpretation of variants was managed according to the American College of Medical Genetics (ACMG) guidelines.

### Sanger Sequencing

All family members were subjected to Sanger sequencing of exon 2 of the *LMOD3* gene. PCR primers were designed by Oligo 6.0 (http://www.oligo.net/downloads.html). Primer sequences and PCR conditions are available upon request.

### Reference Sequence and Variant Nomenclature

NM_198271.4 was employed as the *LMOD3* mRNA reference sequence. Nomenclature for the description of *LMOD3* variants and genotypes followed the Human Genome Variation Society (HGVS) recommendations (den Dunnen et al., 2016).

### Currently Reported Variants in the *LMOD3* Gene

Currently reported variants in the *LMOD3* gene were obtained from the Professional version of the Human Gene Mutation Database (HGMD; https://www.qiagenbioinformatics.com/products/human-gene-mutation-database/) (Stenson et al., 2017) and augmented by a manual literature search.

## Results

Karyotyping of fetus II:3 indicated the presence of a normal set of chromosomes. Chromosomal microarray analysis also failed to identify any pathogenic copy number variants. We then screened II:3 and his parents by WES to search for putative causal variants in an unbiased and hypothesis-free manner. Variants detected were then prioritized based on sequencing quality, allele frequency in the normal population, gene product damage potential, zygosity and mode of inheritance. The mean depth of coverage for the coding regions targeted with the WES was 267×. A mean of 99.2% of bases in the targeted coding regions were covered by at least 10 reads and 99.0% of bases in the targeted coding regions were covered by more than 20 reads. Compound heterozygous truncating variants, c.1283dupC (p.Gly429Trpfs*35) and c.1412delA (p.Lys471Serfs*18), were identified in exon 2 of the *LMOD3* gene (NM_0198272) in II:3; c.1283dupC was inherited from the mother whereas c.1412delA was inherited from the father (**Figure 1A**). The genotypes in II:3 and his parents were confirmed by Sanger sequencing of exon 2 of the *LMOD3* gene. We also sequenced exon 2 of the *LMOD3* gene in II:1 and II:2. Neither variant was found in the former while both variants were found in the latter (**Figure 1A**). Neither c.1283dupC nor c.1412delA have been previously described in the literature and are also absent from the Genome Aggregation Database (gnomAD, http://gnomad.broadinstitute.org/).

Homozygous or compound heterozygous variants (predominantly truncating and nonsense) in the *LMOD3* gene have recently been identified to be a cause of autosomal recessive NM (Yuen et al., 2014). Our identification of novel compound heterozygous truncating variants in the *LMOD3* gene in the two fetuses with polyhydramnios therefore firmly established the diagnosis of NM.

All *LMOD3* variants so far reported to be causative for severe NM are illustrated in the context of the LMOD3 protein structure (**Figure 1B**). The structure of LMOD3 is in accordance with Chereau et al. (2008), UniProt (http://www.uniprot.org/) and InterProScan 4 (Zdobnov and Apweiler, 2001). Additionally, we collated prenatal features in all 22 known *LMOD3* mutation-positive severe NM cases (**Table 1**).

## Discussion

Employing WES, we have successfully identified two novel compound heterozygous truncating variants, c.1283dupC and c.1412delA, in the *LMOD*3 gene in two successive fetuses from the same family with polyhydramnios. *LMOD3* encodes a member of the leiomodin family of proteins, leiomodin-3, which is localized to the pointed ends of thin filaments and acts as a catalyst of actin nucleation in skeletal and cardiac muscle where it is expressed (Conley et al., 2001; Chereau et al., 2008; Qualmann and Kessels, 2009; Campellone and Welch, 2010; Tsukada et al., 2010; Cenik et al., 2015). In 2014, Yuen et al. (2014) identified *LMOD3* as a new causative gene for autosomal recessive NM. NM is a disorder that is characterized by muscle dysfunction and electron-dense protein accumulations (nemaline bodies) in myofibers (Sandaradura and North, 2015). Patients with mutations in *LMOD3* often present with a severe congenital form of early-onset generalized muscle weakness and hypotonia with respiratory insufficiency and feeding difficulties; they usually die in early infancy (Yuen et al., 2014). NM is clinically and genetically heterogeneous, making it difficult to establish a correct diagnosis from clinical features alone. Fetuses with NM usually display decreased fetal movements and polyhydramnios without any particular disease-defining features. The identification of compound heterozygous *LMOD3* variants in our family therefore provided a definite diagnosis of the disease that could not otherwise have been made merely on the basis of clinical findings in the fetus.

To date, a total of 25 *LMOD3* variants have been reported in the literature (**Supplementary Table S1**). Of these, 4 missense variants, p.Arg83His, p.Glu142Asp, p.Lys282Glu, p.Pro552His, have been reported to cause Kleine-Levin syndrome (Al Shareef et al., 2019); a further two missense variants, p.Leu550Phe and p.Gln335Arg, have been associated with a mild form of congenital NM (Schatz et al., 2018) whereas a truncating variant, c.112delG (p.Glu38Lysfs*15), has been detected in a case without a definite clinical diagnosis (Theunissen et al., 2018). By contrast, the remaining 18 variants (i.e., variants highlighted in black in **Figure 1B**) were reported to be causative for severe NM (Yuen et al., 2014; Abbott et al., 2017; Berkenstadt et al., 2018; Michael et al., 2019). Notably, 83% (n = 15) of these latter 18 variants represent truncating variants (nonsense or frameshift). The addition of our two newly identified truncating variants increased this figure to 85% (17/20). Moreover, most previous studies analyzed patients with a diagnosis or suggestive diagnosis of NM (Yuen et al., 2014). Only very recently have *LMOD3* variants been described in any detail in fetuses (Berkenstadt et al., 2018). Our study is the first to describe the prenatal and postnatal manifestations of *LMOD3*-related NM in the Chinese population.

LMOD3 contains three actin-binding domains, which are actin-binding helix [A-h], residues 69–79; leucine-rich repeat domain [LLR], residues 237–402; and Wiskott-Aldrich-syndrome protein homology 2 domain [WH2], residues 534–553 (Yuen et al., 2014). It should be noted that 1 of our 2 novel *LMOD3* mutations, c.1412delA, led to the shortest C-terminal truncation among the *LMOD3* nonsense and frameshifting variants reported to date (**Figure 1B**). This, together with the fact that c.1412delA is associated with a severe form of NM, serves to highlight the indispensable role of the WH2 domain in LMOD3 protein and function. Current models and *in vitro* assays support the view that the WH2 domain, an actin nucleator, stabilizes actin monomers in the trimer conformation, thereby promoting nucleation (Qualmann and Kessels, 2009; Cenik et al., 2015).

Finally, we evaluated the prenatal features of *LMOD3*-related severe NM reported elsewhere (Yuen et al., 2014; Abbott et al., 2017; Berkenstadt et al., 2018; Michael et al., 2019) as well as in this study. As shown in **Table 1**, the most frequently observed anomaly is polyhydramnios (79.2%), followed by decreased fetal movements (70.8%), arthrogryposis/contracture (54.1%), fetal edema (25.0%), and fractures (20.8%). This survey identified fetal edema, which has an even higher frequency than fracture (Abbott et al., 2017), to be a new noteworthy feature of *LMOD3*-related severe NM in the fetal period. Fetal edema could be explained as follows: decreased fetal movement allows accumulation of subcutaneous fluid and causes subsequent atony in the diaphragm, leading to a change in thoracic pressure and a disturbance of lymphatic circulation (Vardon et al., 1998). Taken together, we suggest that *LMOD3-*related severe NM should be considered in cases of prenatal diagnosis of fetal edema and polyhydramnios.

This is the first report of NM-causative *LMOD3* variants, and the first example of prenatal diagnosis of NM, in the Chinese population. The identification of a novel *LMOD3* variant (i.e., c.1412delA) generating the shortest LMOD3 truncation reported to date underscores the critical role of the WH2 domain in LMOD3 structure and function. Further, survey of the prenatal phenotypes of *LMOD3*-related severe NM identified fetal edema as a new presenting feature that may provide an early clue to facilitate prenatal diagnosis of the disease. Finally, we demonstrate once again the power of WES as an effective means to deliver a timely molecular diagnosis in a genetically and clinically highly heterogeneous disorder. We anticipate that the availability of genetic testing for NM will also allow clinicians to offer more reliable prognostic and recurrence risk information to families at risk.

## Data Availability

The datasets used and/or analyzed during the current study are available from the corresponding author on reasonable request.

## Ethics Statement

Written informed consent was obtained from the parents. The study was approved by the Ethics Committee of the First Affiliated Hospital of Sun Yat-Sen University, Guangzhou, China and performed in accordance with the principles enshrined in the Declaration of Helsinki.

## Author Contributions

YW and YZ designed the study with the assistance of J-MC. YW performed the genetic analysis and bioinformatics evaluations. CZ, LD, QL, and M-FL conducted the clinical and ultrasound evaluations. YW and J-MC drafted the manuscript. DC critically revised the manuscript. All authors analyzed the data, contributed to revision, and approved the final manuscript.

## Funding

This work was supported in part by the National Natural Science Foundation of China (no. 31801045) and the PhD Start-up Fund of Natural Science Foundation of Guangdong Province, China (2018A030310244).

## Conflict of Interest Statement

The authors declare that the research was conducted in the absence of any commercial or financial relationships that could be construed as a potential conflict of interest.

## Abbreviations

AFI, amniotic fluid index; GW, gestational weeks; NM, nemaline myopathy; SDP, single deepest pocket; WES, whole exome sequencing.
