# Supplementary material for: Compound Heterozygosity for Novel Truncating Variants in the LMOD3 Gene as the Cause of Polyhydramnios in Two Successive Fetuses
Source: Front Genet. 2019 Sep 13;10:835. doi: 10.3389/fgene.2019.00835 (PMC6753228; doi:10.3389/fgene.2019.00835)
Supplement: Supplementary file 1 [file Table_1.docx]

**Supplementary Table S1. Summary of the 25 known *LMOD3* variants**

| No. | Exon | Nucleotide change | Amino acid change | Type | Disease | Reference |
| --- | --- | --- | --- | --- | --- | --- |
| 1 | 2 | c.112delG | p.Glu38Lysfs*15 | Frame shift | Neuromuscular disorder | Theunissen et al. 2018 |
| 2 | 2 | **c.138dupC** | **p.S47fs*13** | **Frame shift** | **Nemaline myopathy** | **Yuen et al. 2014, Abbott et al. 2017** |
| 3 | 2 | **c.154delA** | **p.M52*** | **Nonsense** | **Nemaline myopathy** | **Yuen et al. 2014** |
| 4 | 2 | **c.231G>A** | **p.W77*** | **Nonsense** | **Nemaline myopathy** | **Yuen et al. 2014** |
| 5 | 2 | c.248G>A | p.R83H | Missense | Kleine-Levin syndrome | Al Shareef et al. 2019 |
| 6 | 2 | **c.300_304delGACTC** | **p.T101Rfs*4** | **Frame shift** | **Nemaline myopathy** | **Yuen et al. 2014** |
| 7 | 2 | **c.349C>T** | **p.Q117*** | **Nonsense** | **Nemaline myopathy** | **Yuen et al. 2014** |
| 8 | 2 | - | **p.E121Rfs*5** | **Frame shift** | **Nemaline myopathy** | **Berkenstadt et al. 2018** |
| 9 | 2 | c.426A>C | p.E142D | Missense | Kleine-Levin syndrome | Al Shareef et al. 2019 |
| 10 | 2 | **c.601_602delGA** | **p.D201Efs*9** | **Frame shift** | **Nemaline myopathy** | **Yuen et al. 2014** |
| 11 | 2 | **c.723_733del** | **p.D242Efs*4** | **Frame shift** | **Nemaline myopathy** | **Yuen et al. 2014** |
| 12 | 2 | - | **p.L245del** | **In-frame** | **Nemaline myopathy** | **Berkenstadt et al. 2018** |
| 13 | 2 | c.844A>G | p.K282E | Missense | Kleine-Levin syndrome | Al Shareef et al. 2019 |
| 14 | 2 | **c.860del** | **p.F287Sfs*3** | **Frame shift** | **Nemaline myopathy** | **Yuen et al. 2014** |
| 15 | 2 | **c.882dupA** | **p.D295Rfs*2** | **Frame shift** | **Nemaline myopathy** | **Michael et al. 201** |
| 16 | 2 | **c.976G>C** | **p.G326R** | **Missense** | **Nemaline myopathy** | **Yuen et al. 2014** |
| 17 | 2 | c.1004A>G | p.Q335R | Missense | Milder nemaline myopathy | Schatz et al. 2018 |
| 18 | 2 | **c.1069G>T** | **p.E357*** | **Nonsense** | **Nemaline myopathy** | **Yuen et al. 2014** |
| 19 | 2 | **c.1099_1100delAA** | **p.N367Qfs*11** | **Frame shift** | **Nemaline myopathy** | **Yuen et al. 2014** |
| 20 | 2 | **c.1100_1102delACA** | **p.N367del** | **In-frame** | **Nemaline myopathy** | **Yuen et al. 2014** |
| 21 | 2 | **c.1201C>T** | **p.R401*** | **Nonsense** | **Nemaline myopathy** | **Yuen et al. 2014** |
| 22 | 2 | **c.1218delA** | **p.K406Nfs*11** | **Frame shift** | **Nemaline myopathy** | **Yuen et al. 2014** |
| 23 | 2 | **c.1372C>T** | **p.Q458*** | **Nonsense** | **Nemaline myopathy** | **Yuen et al. 2014** |
| 24 | 2 | c.1648C>T | p.L550F | Missense | Milder nemaline myopathy | Schatz et al. 2018 |
| 25 | 2 | c.1655C>A | p.P552H | Missense | Kleine-Levin syndrome | Al Shareef et al. 2019 |

NM_198271.4 was employed as the *LMOD3* mRNA reference sequence. Variants highlighted in bold correspond to those illustrated in Figure 1B.
